# Insight Into Spinocerebellar Ataxia Type 31 (SCA31) From *Drosophila* Model

**DOI:** 10.3389/fnins.2021.648133

**Published:** 2021-05-25

**Authors:** Taro Ishiguro, Yoshitaka Nagai, Kinya Ishikawa

**Affiliations:** ^1^Department of Neurology and Neurological Science, Tokyo Medical and Dental University, Bunkyo City, Japan; ^2^Department of Neurotherapeutics, Osaka University Graduate School of Medicine, Suita, Japan; ^3^Department of Personalized Genomic Medicine for Health, Graduate School, Tokyo Medical and Dental University, Bunkyo City, Japan

**Keywords:** RBP, TDP-43, RNA chaperone, RNA foci, RAN translation, microsatellite repeat, SCA31

## Abstract

Spinocerebellar ataxia type 31 (SCA31) is a progressive neurodegenerative disease characterized by degeneration of Purkinje cells in the cerebellum. Its genetic cause is a 2.5- to 3.8-kb-long complex pentanucleotide repeat insertion containing (TGGAA)n, (TAGAA)n, (TAAAA)n, and (TAAAATAGAA)n located in an intron shared by two different genes: brain expressed associated with NEDD4-1 (*BEAN1*) and thymidine kinase 2 (*TK2*). Among these repeat sequences, (TGGAA)n repeat was the only sequence segregating with SCA31, which strongly suggests its pathogenicity. In SCA31 patient brains, the mutant BEAN1 transcript containing expanded UGGAA repeats (UGGAA_exp_) was found to form abnormal RNA structures called RNA foci in cerebellar Purkinje cell nuclei. In addition, the deposition of pentapeptide repeat (PPR) proteins, poly(Trp-Asn-Gly-Met-Glu), translated from UGGAA_exp_ RNA, was detected in the cytoplasm of Purkinje cells. To uncover the pathogenesis of UGGAA_exp_ in SCA31, we generated *Drosophila* models of SCA31 expressing UGGAA_exp_ RNA. The toxicity of UGGAA_exp_ depended on its length and expression level, which was accompanied by the accumulation of RNA foci and translation of repeat-associated PPR proteins in *Drosophila*, consistent with the observation in SCA31 patient brains. We also revealed that TDP-43, FUS, and hnRNPA2B1, motor neuron disease–linked RNA-binding proteins bound to UGGAA_exp_ RNA, act as RNA chaperones to regulate the formation of RNA foci and repeat-associated translation. Further research on the role of RNA-binding proteins as RNA chaperones may also provide a novel therapeutic strategy for other microsatellite repeat expansion diseases besides SCA31.

## Introduction

More than 40 refractory neurological diseases are caused by microsatellite repeat expansions, including spinal and bulbar muscular atrophy; Huntington disease (HD); Friedreich ataxia; fragile X syndrome; fragile X–associated tremor/ataxia syndrome (FXTAS); myotonic dystrophy types 1 and 2 (DM1, DM2); C9orf72-associated amyotrophic lateral sclerosis/frontotemporal dementia (C9orf72-ALS/FTD); dentatorubral-pallidoluysian atrophy; spinocerebellar ataxia (SCA) types 1, 2, 3, 6, 7, 8, 10, 12, 17, 31, 36, and 37 (Paulson, 2018); and benign adult familial myoclonic epilepsy (Ishiura et al., 2018). Microsatellite repeats are tandem stretches of three to six nucleotides in the genome that are often polymorphic in length. At some genetic loci, microsatellite repeats become genetically unstable (Pearson et al., 2005; Nelson et al., 2013; Banez-Coronel and Ranum, 2019) and cause disease when the length of the repeat sequence exceeds a certain threshold. This size threshold differs for each repeat-harboring gene. In affected individuals, large repeat expansions show somatic and intergenerational instabilities and lead to various disease phenotypes. Disease-causing repeat expansion mutations can reside in both coding and noncoding regions of the respective gene, including the promoter region, 5′ UTR, an alternate exon, 3′ UTR, and an intron. The lengths of repeat expansion in noncoding regions are usually much greater and more unstable than those in coding regions. The molecular pathogenesis of these repeat expansion diseases is considered to include loss of host gene function as in fragile X syndrome and Friedreich ataxia, production of toxic RNAs as in DM1 and C9orf72-ALS/FTD, and production of toxic polypeptides as in polyglutamine diseases such as HD and several SCAs. The complexity of their pathogenesis has gradually been recognized: the loss of host gene function and the production of both toxic RNA and polypeptides all seem to play roles in a single disease, as exemplified by the pathogenesis of HD. The repeat expansions in some of these diseases are transcribed into aberrant repeat RNAs, which can form hairpin and G-quadruplex structures and RNA foci that interact with RNA-binding proteins (RBPs) and alter their activities, which would lead to dysfunction in RNA metabolism (Mohan et al., 2014; Liu et al., 2017; Zhang and Ashizawa, 2017; Reddy et al., 2020). For example, the depletion of key splicing regulatory RBPs such as muscleblind-like 1 (MBNL1) and CUG-binding protein 1 (CUGNP1) sequestered in RNA foci can induce pre-mRNA missplicing in DM1. Furthermore, expanded repeat RNAs undergo repeat-associated non-ATG (RAN) translation to generate toxic repeat proteins in all reading frames (Zu et al., 2011, 2013). Taking these findings together, the microsatellite repeat expansions trigger a wide range of pathogenic pathways, which leads to a variety of neurological and neurodegenerative diseases.

## Overview of SCA31: Clinical Perspective and Features

SCA31 is one of the autosomal-dominant neurodegenerative disorders showing a relatively pure cerebellar form of ataxia. It is typically a disease of late adulthood, with onset peaking between 60 and 65 years of age (Nagaoka et al., 2000; Sato et al., 2009; Nakamura et al., 2017; Ishikawa and Nagai, 2019). SCA31 is a common ataxia in Japan (Onodera, 2006; Ouyang et al., 2006; Basri et al., 2007; Hayashi et al., 2007; Nozaki et al., 2007; Yoshida et al., 2009), whereas it is very rare in surrounding parts of Asia such as Korea, Taiwan, and China (Lee et al., 2007, 2012; Ouyang et al., 2012; Pedroso et al., 2015) and extremely rare in Caucasian populations (Ishikawa et al., 2011). SCA31 patients were previously found in Brazil, but all of them were descendants of Japanese immigrants (Pedroso et al., 2015), which suggests that SCA31 shows a strong founder effect.

**FIGURE 1:**
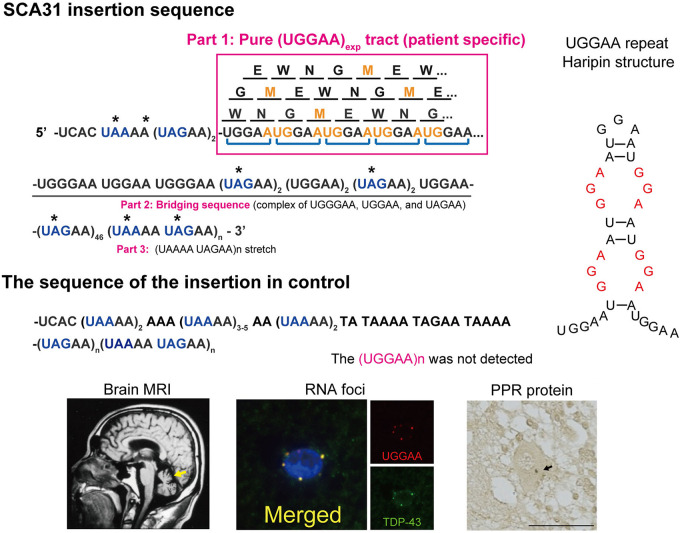
Schematic diagram of PPR proteins. Amino acids are shown as single letters. Translation of the UGGAA repeat in all frames would result in the production of an identical PPR protein, poly-WNGME. Asterisk (*) indicates a stop codon in the sequence surrounding the UGGAA repeat. Note that the TGGAA repetitive sequence itself includes an AUG translation–initiation codon, and both the TAAAA and the TAGAA sequences flanking the TGGAA repetitive sequence include UAA and UAG translation stop codons. AUG codons shown in orange stand for start, and blue color codons stand for stop in repeat sequence in a *BEAN1* transcript. SCA31 shows slowly progressive cerebellar ataxia and MRI-proven cerebellar atrophy. Purkinje cells are primarily affected showing characteristic degenerative changes. Abnormal RNA aggregates called RNA foci are occasionally detected in Purkinje cell nuclei by fluorescent *in situ* hybridization (FISH) using probes against the (UGGAA)n-containing transcripts (Ishiguro et al., 2017). Furthermore, TDP-43 clearly colocalized with RNA foci in human SCA31 Purkinje cells. PPR proteins were also detected in cell bodies and dendrites of SCA31 Purkinje cells (arrows) (Ishiguro et al., 2017). Possible hairpin structures in UGGAA repeat containing internal loop with three consecutive G-A, G-G, and A-G mismatch base pairs (shown in red) (Shibata et al., 2021).

SCA31 is caused by a 2.5- to 3.8-kb insertion of complex pentanucleotide repeats containing (TGGAA)n, which is located in overlapping introns of the *BEAN1* and *TK2* genes on chromosome 16q22.1 (Sato et al., 2009). The insertion sequence is a complex pentanucleotide repeat containing (TGGAA)n, (TAGAA)n, (TAAAA)n, and (TAAAATAGAA)n (Figure 1). Importantly, the length of this insertion was shown to be inversely correlated with the age at onset in SCA31 patients. Among healthy controls, the vast majority (99.7%) were found to have a short TAAAA repeat of only 8–20 repeats at this locus, although very rare large insertions of (TAAAA)n, (TAGAA)n, and (TAAAATAGAA)n stretch (approximately 1.5–2.0 kb in size) lacking (TGGAA) sequences were found in Japanese controls (0.23%). Therefore, (TGGAA)n was considered to be the only causative repetitive sequence among these repeats segregating with the phenotype in SCA31. In addition to these repeats, the SCA31 mutation site is associated with diverse pentanucleotide repeats such as (TACAA)n, (GAAAA)n, (TAACA)n, (TGAAA)n, and (TAAAA)n. While (TAAAA)n is the prototypical pentanucleotide repeat in humans, (TGGAA)n and (TAGAA)n repeats are exclusively found in the Japanese, whereas (TACAA)n, (GAAAA)n, (TAACA)n, and (TGAAA)n pure repeat expansions are found in Caucasians (Ishikawa et al., 2011). Among these repeats, (TGGAA)n is the only repetitive sequence that segregates with cerebellar neurodegeneration.

## Neuropathological Features of SCA31

In patients with SCA31, marked degeneration of the cerebellar cortex with Purkinje cell–predominant neuronal loss was observed in the cerebellum (Owada et al., 2005). Purkinje cells often showed shrinkage of the cell body. In addition, they were often surrounded by halo-like amorphous materials, which were shown to be positively stained with anti–calbindin-D-28 K (CaBP28K) and anti-synaptophysin antibodies in immunohistochemistry (Ishikawa and Mizusawa, 2010; Yoshida et al., 2014). Therefore, these materials were considered to consist mainly of the somatic sprouts of Purkinje cells and presynaptic terminals innervated from basket cells, neurons of the inferior olivary nucleus, or other neurons that connect with Purkinje cells.

A neuropathological hallmark of microsatellite repeat expansion diseases is the intracellular accumulation of mutant RNA containing an expanded nucleotide repeat as RNA foci in the nucleus and/or cytoplasm of affected cells. This has emerged as an important disease mechanism for the growing class of such diseases (Wojciechowska and Krzyzosiak, 2011; Zhang and Ashizawa, 2017). Previous studies identified RNA foci within the nuclei of SCA31 Purkinje cells labeled with an oligonucleotide probe against UGGAA repeats (Sato et al., 2009; Niimi et al., 2013).

Several lines of evidence indicate that RAN translated proteins are involved in a growing number of microsatellite repeat expansion diseases, including HD, DM1, SCA8, FXTAS, and C9orf72-ALS/FTD (Banez-Coronel and Ranum, 2019; Nguyen et al., 2019). Unlike other microsatellite repeat expansion diseases, it is important to note that, in SCA31, the UGGAA repetitive sequence itself includes an AUG translation–initiation codon (Ishiguro et al., 2017). On the other hand, several repetitive sequences in the SCA31 locus found in normal populations, including UAAAA, UAGAA, UGAAA, and UAACA, contain UAA, UAG, and UGA translation stop codons, and thus these repeats are unlikely to be translated. Translation of the UGGAA repeat in any frame would result in a single pentapeptide repeat (PPR) protein, poly-WNGME. Another special circumstance in SCA31 is that the UGGAA repeat is flanked by putative stop codons UAA and UAG (Figure 1), which suggests that, if the PPR is expressed, the translation has to occur within this sequence. In fact, anti-PPR antibodies detected granular structures in cell bodies and dendrites of SCA31 Purkinje cells, but not in control brains, which suggests that PPR proteins are expressed in SCA31 brains where (UGGAA)_exp_ is expressed by *BEAN1* transcription. Taking these findings together, TGGAA pentanucleotide repeat expansion gives rise to toxic UGGAA repeat RNAs, which form RNA foci and are translated into poly(WNGME) proteins in Purkinje cells in SCA31.

Much of the previous progress in our understanding of various microsatellite repeat expansion diseases suggests the multifaceted nature of the underlying pathogenic mechanisms in SCA31. The first pathogenic mechanism of SCA31 suggested by nuclear and cytoplasmic RNA foci containing UGGAA_exp_ transcripts is RNA-mediated toxicity, as these foci might sequester RNA-binding proteins and consequently alter RNA metabolism, as in other repeat expansion disorders (Sato et al., 2009; Niimi et al., 2013; Ishiguro et al., 2017). Another distinctive feature of SCA31 is the deposition of PPR proteins, mostly in the cytoplasm of patients’ Purkinje cells: poly(WNGME) is synthesized from the UGGAA repeat transcripts. Repeat-associated AUG or non-AUG (RAN) translation is proposed to be the mechanism responsible for PPR protein production (Ishiguro et al., 2017). Note that the TGGAA repetitive sequence itself includes an AUG translation–initiation codon. Haploinsufficiency of the causative gene is also a potential disease-causing mechanism in microsatellite repeat expansion diseases; however, the expression of *BEAN1*, *TK2*, and nearby genes in SCA31 patient brains is not reduced by the insertion of complex repeats in this locus, which indicates that haploinsufficiency is unlikely to be a primary mechanism behind the disease. The expanded repeat can be transcribed in both sense and antisense directions, resulting in sense and antisense RNA foci or protein aggregates in several repeat expansion diseases including SCA8, HDL2, and C9ALS/FTD (Moseley et al., 2006; Wilburn et al., 2011; Gendron et al., 2013; Zu et al., 2013). Considering that the insertion is located in introns of *BEAN1* and *TK2* transcribed in opposite directions in SCA31, *TK2* transcripts including extended repeats may behave in a similar manner. The function of this transcript and its toxicity remains to be explored. To elucidate the molecular mechanisms of SCA31, we employed *Drosophila* to model SCA31 expressing UGGAA repeat in *BEAN1* direction as it has been productively used to study neurodegenerative diseases in recent years (McGurk et al., 2015; Şentürk and Bellen, 2018). Insights from *Drosophila* models of SCA31 are discussed below.

## *Drosophila* as a Genetic Model for SCA31

We have established *Drosophila* models of SCA31 expressing expanded UGGAA repeats to analyze the gain of toxic function, in a manner mostly similar to that used for other repeat expansion diseases (Ishiguro et al., 2017). Eighty to 100 copies of TGGAA repeats together with three other repeat components, (TAGAA)n, (TAAAA)n, and (TAAAATAGAA)n, derived from SCA31 patients were subcloned into the pUAST vector, which has an upstream activating sequence (UAS) element to allow spatial control of target gene expression in tissue-specific GAL4 driver lines, and transgenic flies carrying TGGAA repeats were generated. Control fly lines were also established expressing control repeat tract lacking (TGGAA)n found in the normal population. In both transiently and stably expressed cultured cell models, the (UGGAA)n in the *BEAN1* transcripts yields more toxicity than control transcripts and forms RNA foci (Niimi et al., 2013). The contribution of the UGGAA repeat tract to disease pathogenesis *in vivo* was first evaluated in the *Drosophila* models of UGGAA_exp_ described above. The expression of UGGAA_exp_ (80–100 repeats) RNA in compound eyes using the GMR-GAL4 driver caused severe eye degeneration, depending on its expression level, whereas control repeat and short UGGAA_22_ RNA showed no detectable degeneration.

The expression of UGGAA_exp_ in the nervous system with elav-Gal4 driver exhibited a shorter lifespan and progressive locomotor defects, whereas control repeat and UGGAA_22_ did not. As expected, RNA fluorescence *in situ* hybridization (FISH) revealed remarkable accumulation of UGGAA_exp_ RNA as RNA foci in eye imaginal disks of UGGAA_exp_-expressing flies, consistent with the pathology of SCA31 patients. Fly lines expressing control repeat or UGGAA_22_ had no detectable RNA foci in the fly tissue. In this model, PPR protein was also detected by Western blot and immunohistochemistry, and its expression level was positively correlated to the severity of eye degeneration in *Drosophila*. Therefore, both UGGAA_exp_ RNA toxicity and PPR protein toxicity may contribute to the neurodegeneration in SCA31.

## TDP-43 Disentangles UGGAA_exp_ RNA From RNA Foci Formation

RNA-binding proteins are essential at all levels of RNA processing, including transcription, alternative splicing, stabilization, degradation, and RNA granule formation, in both the nucleus and the cytoplasm. The loss of RBPs influences the processing of these noncoding RNAs and contributes to global RNA dysregulation. Considering that proteins sequestered in RNA foci have been reported to play an important role in gain of toxic function of microsatellite repeat expansion diseases (Zhang and Ashizawa, 2017), the interacting partners of repeat RNA have been studied extensively in SCA31 using pull-down approaches (Ishiguro et al., 2017).

**FIGURE 2:**
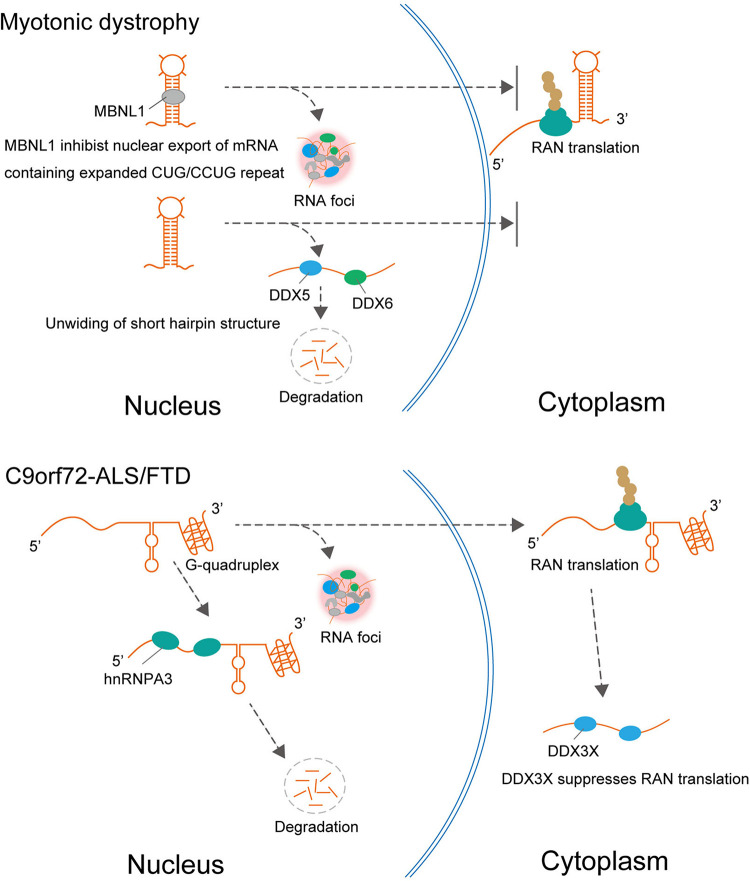
In DM1, MBNL1 promotes nuclear retention of expanded CUG repeat RNA and RNA foci formation, resulting in repression of RAN translation from CUG repeat RNA. In addition to MBNL1, DDX5, and DDX6 are also involved in DM1. DDX5 leads to degradation of the expanded CUG and CCUG RNAs and suppresses RNA foci formation. In C9orf72-ALS/FTD, hnRNPA3 reduced repeat RNA expression levels leading to decreasing RNA foci formation and DPR deposition. The helicase DDX3X, which unwinds (or relaxes) RNA, suppresses RAN translation and toxicity.

**FIGURE 3:**
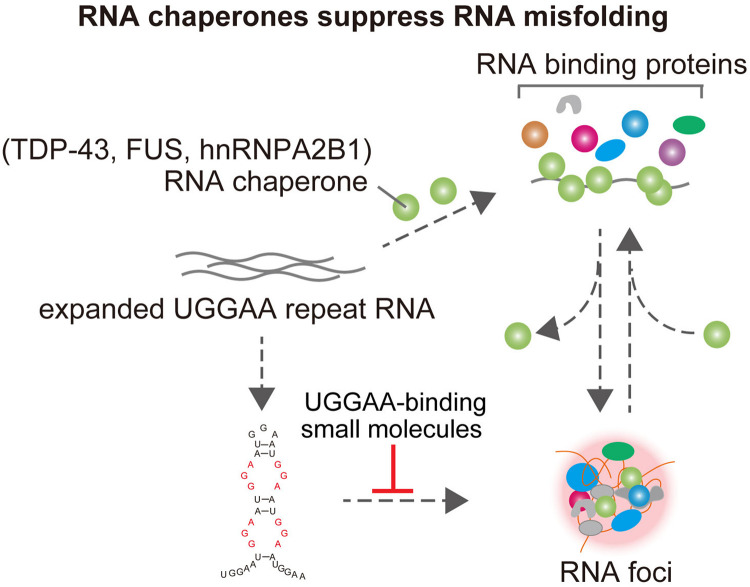
TDP-43 has RNA chaperone activity, structurally altering RNAs and affects UGGAA repeat-mediated toxicity and RNA foci formation in SCA31. FUS and hnRNPA2B1 also bind do UGGAA repetitive RNA and act as RNA chaperones, suppressing RNA foci formation and PPR translation. Small-molecule binders to UGGAA repeat inhibit RNA foci formation.

Interestingly, several ALS/FTD-linked RBPs were identified as UGGAA-binding proteins, such as TDP-43 (TAR DNA-binding protein, 43 kDa), FUS, and hnRNPA2B1. Indeed, FISH analysis showed the colocalization of TDP-43 with RNA foci in the nucleus of human SCA31 Purkinje cells. Surprisingly, when TDP-43 was coexpressed with UGGAA_exp_ in *Drosophila*, the UGGAA_exp_-mediated eye degeneration and accumulation of RNA foci were dramatically suppressed. Conversely, the knockdown of TDP-43 enhanced eye degeneration and the formation of RNA foci. Subsequent analysis of atomic force microscopy and circular dichroism spectroscopy demonstrated that the binding of TDP-43 to UGGAA repeat RNA alters its structure. Thus, TDP-43 functions as an RNA chaperone for UGGAA repeat RNA, consistent with the definition of an RNA chaperone (Figure 2; Rajkowitsch et al., 2007). Notably, as mentioned earlier, PPR proteins are also expressed in *Drosophila* tissue and SCA31 brain. TDP-43 coexpression with expanded UGGAA in the fly was shown to dramatically inhibit the expression and accumulation of PPR protein in fly tissue. In addition to TDP-43, coexpression of FUS or hnRNPA2B1 markedly suppressed eye degeneration in UGGAA_exp_-expressing flies. These RBPs mitigated the deposition of both RNA foci and PPR proteins without causing degradation of the UGGAA_exp_ RNA transcripts, confirming their function as RNA chaperones. TDP-43 and hnRNPA2B1 also have a protective role against CGG-repeat mediated toxicity in *Drosophila* model of FXTAS (He et al., 2014). Interestingly, it is suggested that TDP-43 suppression of CGG repeat toxicity is dependent on interaction with hnRNPA2B1 orthologs in an FXTAS *Drosophila* model. Overexpression of TDP-43 suppresses CGG repeat-mediated missplicing of hnRNPA2/B1-targeted transcripts. Note that TDP-43, FUS, and hnRNPA2B1 act independently to suppress UGGAA repeat-mediated toxicity in *Drosophila* model of SCA31. These results suggest that the rescuing effect of TDP-43 for FXTAS could be different from that for SCA31. Purα is an RBP identified to interact with CGG repeat in *Drosophila* model of FXTAS (Jin et al., 2007). Overexpression of Purα suppresses CGG repeat-mediated neurodegeneration in *Drosophila*. Importantly, Purα is also found in the nuclear inclusions of FXTAS patient brains. These findings suggest that RNA-binding proteins that associate with repeat RNAs play an important role in the pathogenesis in repeat expansion diseases. Targeting factors critical for modulating RNA secondary structures could be potential therapeutic strategy in FXTAS and C9ALS/FTD as well. Knockdown of DDX3X, Dead-box helicase that is required for resolution of RNA-RNA structures and 43S PIC-mRNA binding and PIC scanning in GC-rich 5′ UTR in *FMR1*, suppresses CGG repeat-mediated toxicity by inhibiting RAN translation (Linsalata et al., 2019). On the other hand, surprisingly, expression of DDX3X reduces endogenous DPR level and mitigates toxicity in C9ALS/FTD patient cells, and silencing of DDX3X enhances GGGGCC repeat-mediated toxicity in *Drosophila* of C9ALS/FTD (Figure 3; Cheng et al., 2019). Although DDX3X has opposing effects on RAN translation of GGGGCC repeat in C9ALS/FTD and CGG repeat in FXTAS, an RNA helicase that unwinds the repeat RNA structure would be expected to reduce RAN translation via modulation of RNA secondary structures. Taken together, targeting factors that modulate RNA secondary structures associated with RNA foci formation and RAN translation could be potential therapeutic strategies for SCA31 and related repeat expansion diseases.

Interestingly, these suppressive effects of TDP-43, FUS, and hnRNPA2B1 differ from those of other RBPs that interact with expanded repeat RNAs such as MBNL1 in DM1 and hnRNPA3 in C9orf72-ALS/FTD (Figure 2). For example, MBNL1 is sequestered in intranuclear inclusions containing expanded repeat RNA in the nucleus in DM1 and DM2, which leads to loss of MBNL function. In this process, MBNL1 promotes the nuclear retention of expanded CUG repeat RNA and the formation of RNA foci, resulting in the repression of RAN translation from CUG repeat RNA (Kino et al., 2015). It is also suggested that hnRNP H inhibits nuclear export of the expanded CUG RNA, which leads to its nuclear retention in DM1 (Kim et al., 2005). Therefore, several RBPs may play a protective role by promoting mutant RNA nuclear retention and the formation of RNA foci, which could act as an RNA sink associated with RBPs. In addition to MBNL1 and hnRNPH, Staufen1 and two members of the DEAD-box RNA helicase family, DDX5 and DDX6, are also involved in DM1. The protein expression level of DDX5 is reduced in DM1 biopsied muscle, and upregulation of DDX5 leads to degradation of the expanded CUG and CCUG RNAs and suppresses the formation of RNA foci (Pettersson et al., 2014; Jones et al., 2015). hnRNPA3 is one member of the heterogeneous nuclear ribonucleoprotein family, which specifically binds to the GGGGCC repeat RNA in the pathology of C9orf72-ALS/FTD (Mori et al., 2013). Unlike the protective role of RNA chaperones in SCA31, hnRNPA3 negatively regulates repeat RNA expression levels, which leads to decreased formation of RNA foci and DPR deposition (Mori et al., 2016).

## Small-Molecule Binders to UGGAA Repeat Inhibit RNA Foci Formation and Suppress Eye Degeneration in *Drosophila* Model of SCA31

GGGGCC repeat RNA folds into a G-quadruplex and hairpin secondary structure in C9ALS/FTD, and CGG repeat RNA forms hairpin structure in FXTAS. Previous intensive research discovered that small molecules, which selectively bound GGGGCC or CGG repeat RNA, prevented sequestration of an RBP and inhibited RAN translation *in vitro* and *in vivo* models of C9ALS/FTD or FXTAS (Su et al., 2014; Simone et al., 2018; Green et al., 2019). These results suggest that targeting RNA secondary structures related to neurodegeneration could be a potential therapeutic strategy. UGGAA repeat is also suggested to fold into hairpin structure containing internal loop with three consecutive G-A, G-G, and A-G mismatch base pairs (Figure 1; Shibata et al., 2021). Recently, a chemical screen identified naphthyridine carbamate dimer (NCD) targeting disease-causing UGGAA repeat RNAs in SCA31 (Shibata et al., 2021). Structural analysis of the NCD-UGGAA/UGGAA complex by nuclear magnetic resonance spectroscopy revealed the mode of binding that recognizes four guanines in the UGGAA/UGGAA pentad by hydrogen bonding with four naphthyridine moieties of two NCD molecules. NCD disrupts RNA foci formation of UGGAA RNA by inhibiting interaction of UGGAA RNA and RBPs *in vitro* and ameliorates compound eye degeneration in the *Drosophila* model of SCA31 (Figure 3). These data provide proof of principle that targeting UGGGAA repeat harpin has therapeutic potential as well as G-quadruplex and hairpin secondary structure in C9ALS/FTD or FXTAS.

## Balancing RNA–RBP Crosstalk Is Essential for RNP Homeostasis

Although our SCA31 studies highlighted novel roles of TDP-43, FUS, and hnRNPA2B1 as RNA chaperones for UGGAA_exp_ RNA to modulate its folding and regulate the formation of toxic RNA aggregates (Figure 3), these RBPs on their own are known to play a toxic role in the pathogenesis of ALS/FTD (Ling et al., 2013). For example, pathological inclusions of TDP-43 can be found in the nucleus and cytosol of neurons and glia in sporadic and familial forms of ALS and FTD. Furthermore, patient-derived induced pluripotent stem cells and overexpression of TDP-43 in experimental animals recapitulated some of the pathological features of human ALS and FTD with neurodegeneration accompanied by various molecular phenotypes such as TDP-43 aggregation and aberrant RNA metabolism. Given that the UGGAA repeat RNA interacts with these RBPs, it is possible that such RBP toxicity may be ameliorated by binding RNAs. Against this background, flies expressing ALS-linked mutant TDP-43 G298S were crossed with UGGAA_22_ flies, which do not show any neurodegeneration. Although TDP-43 G298S-expressing flies exhibited severe compound eye degeneration, coexpression of UGGAA_22_ significantly suppressed the eye degeneration as well as mutant TDP-43 aggregation in eye imaginal disks. Furthermore, coexpression of UGGAA_22_ also suppressed the eye degeneration of flies expressing either FUS or mutant hnRNPA2B1 D290V, which suggests that UGGAA_22_ buffers the propensity of these RBPs to aggregate (Ishiguro et al., 2017). These results reveal that functional crosstalk of the RNA–RBP network regulates their own quality and homeostasis of ribonucleoprotein (RNP), which suggests that balancing the RNA–RBP crosstalk is a potential therapeutic approach for both microsatellite repeat expansion diseases and RBP proteinopathies.

Recent intensive studies have suggested that RBPs containing low complexity domains such as TDP-43, FUS, and hnRNPA2B1 can induce liquid–liquid phase separation in the process of formation of dynamic membrane-less organelles including Cajal bodies, paraspeckles, nuclear speckles, and stress granules (Weber and Brangwynne, 2012; Molliex et al., 2015; Courchaine et al., 2016). The combination of protein subdomains; RNA parameters such as concentration, structure, length, and sequence composition; and physiological salt conditions can greatly influence phase separation (Kroschwald et al., 2015; Jain and Vale, 2017; Maharana et al., 2018). Increasing the concentration of RBPs can diminish the liquid-like properties of these RNA granules, thereby promoting the formation of hydrogels and eventually an insoluble amyloid-like aggregate (Kato et al., 2012; Guo and Shorter, 2015; Shin and Brangwynne, 2017; Wolozin, 2019; Rhine et al., 2020). Conversely, the high RNA-to-protein concentration ratio in the nucleus buffers the phase separation of RBPs such as FUS and TDP-43 (Gasset-Rosa et al., 2019). Taking these findings together, considering that balancing RNA–RBP crosstalk is essential for RNP homeostasis, both the RNA chaperone activity of RBPs and the buffering function of RNAs for target RBPs might be essential to regulate the formation of membrane-less organelles through proper liquid–liquid phase separation and the maintenance of these compartments.

## UGGAA_exp_ Is a Common Modular Structure With Satellite III RNAs in the Formation of nSBs

From an *in silico* search of TGGAA pentanucleotide sequences in the human genome, TGGAA repeats are abundant in the centromeres of chromosomes 2, 4, 7, 10, 16, 17, 20, and Y. In addition, satellite III (SatIII) DNA in pericentromeric heterochromatin also has a modular structure composed of the TGGAA pentanucleotide repeats in common with SCA31 (Valgardsdottir et al., 2004). Notably, SatIII noncoding RNA containing UGGAA repeats is transcribed under heat stress to form nuclear stress bodies (nSBs) and plays an important role in regulating the splicing machinery by recruiting certain splicing factors such as serine/arginine-rich splicing factor 1 (SRSF1) and scaffold attachment factor B to nSBs (Metz et al., 2004; Valgardsdottir et al., 2008; Eymery et al., 2010). The recruitment of SRSF1 to nSBs occurs in a SatIII-dependent manner, and the downregulation of SatIII transcripts attenuates the heat shock–induced transcriptional repression of target genes. Conversely, the overexpression of SATIII transcripts results in the formation of nSBs and transcriptional repression, even without heat shock. Considering that SFRS1 and SFRS9 directly bind to UGGAA_exp_, the expression of UGGAA_exp_ in SCA31 brain may competitively inhibit SatIII function, which leads to the misregulation of nSB formation and stress response. Indeed, the neuron-specific knockdown of *Drosophila* hsrω, functional analogs of SatIII, impairs locomotion in larval and adult flies, which implies that the dysfunction of SatIII mediated by UGGAA_exp_ RNA in SCA31 could lead to neurodegeneration (Jolly and Lakhotia, 2006; Lo Piccolo and Yamaguchi, 2017; Lo Piccolo et al., 2019).

## Concluding Remarks

In this article, we reviewed recent progress in our understanding of the pathogenesis of SCA31, mainly focusing on our recent findings revealed by *Drosophila* models. We demonstrated a novel role of repeat RNA-binding RBPs as RNA chaperones that modulate the repeat RNA misfolding and buffer its toxicity (Figure 3; Ishiguro et al., 2017).

**FIGURE 4:**
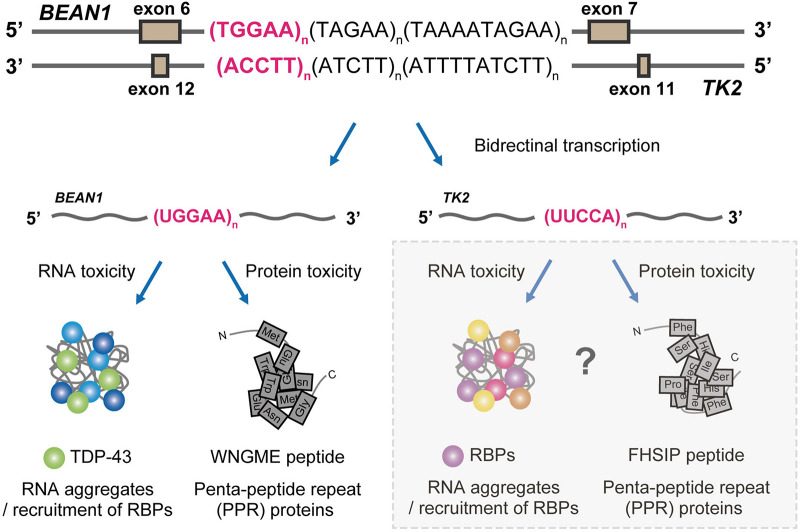
Proposed pathogenic mechanisms in SCA31. BEAN1 transcripts including expanded UGGAA repeat and TK2 transcripts including expanded UUCCA repeat may cause neurodegeneration by RNA foci formation and RAN translation. Further investigation will clarify whether TK2 transcripts with the expanded repeat behave in a similar manner.

Our study not only provided novel insights into the molecular pathogenesis of SCA31 and related repeat expansion diseases but also opened a new avenue toward the development of therapies for these diseases. Nevertheless, the presence of both RNA foci and PPR proteins in SCA31 still raises important questions about their relative contributions to the disease pathogenesis, and several questions still remain unanswered: (i) How can UGGAA_exp_ and RNA foci induce toxicity leading to neurodegeneration? (ii) Are PPR proteins necessary or sufficient to induce neurotoxicity? (iii) Do RBPs that bind to other disease-causing repetitive sequences such as C9orf72-ALS/FTD, FXTAS, SCA36, or DM1 indeed play a protective role as RNA chaperones? (iv) Can *TK2* transcripts including extended UUCCA repeats cause neurodegeneration with RNA foci formation and RAN translation? (Figure 4). It is important to determine whether UUCCA repeats could form RNA foci and sequester RBPs and produce poly(Phe-His-Ser-Ile-Pro, FHSIP) peptide in SCA31. We hope that genetic approaches taking advantage of *Drosophila* will help to address these important issues and contribute to establishing therapies for SCA31 and other repeat expansion diseases.

## Author Contributions

TI, YN, and KI designed the manuscript, assessed the literature, and edited the subsequent drafts and revisions. TI wrote the initial draft of the manuscript. All the authors contributed to the article and approved the submitted version.

## Conflict of Interest

The authors declare that the research was conducted in the absence of any commercial or financial relationships that could be construed as a potential conflict of interest.
